# Structure-mutagenicity relationships on quinoline and indole analogues in the Ames test

**DOI:** 10.1186/s41021-024-00316-6

**Published:** 2024-11-14

**Authors:** Masaki Kurakami, Atsushi Hakura, Rika Sato, Akihiro Kawade, Takeshi Yamagata, Naoki Koyama, Dai Kakiuchi, Shoji Asakura

**Affiliations:** 1 Global Drug Safety, Eisai Co., Ltd., 5-1-3 Tokodai, Tsukuba-shi, Ibaraki 300-2635 Japan; 2 Tsukuba Division, Sunplanet Co., Ltd., 5-1-3 Tokodai, Tsukuba-shi, Ibaraki 300-2635 Japan; 3 Quality Assurance Department, Sunplanet Co., Ltd., 5-1-3 Tokodai, Tsukuba-shi, Ibaraki 300-2635 Japan

**Keywords:** Ames test, Mutagenicity, Heterocyclic compounds, Quinoline, Indole, Structure-mutagenicity relationship, In silico

## Abstract

**Background:**

Although the in silico predictive ability of the Ames test results has recently made remarkable progress, there are still some chemical classes for which the predictive ability is not yet sufficient due to a lack of Ames test data. These classes include simple heterocyclic compounds. This study aimed to investigate the mutagenicity and structure-mutagenicity relationships for some heterocycles in the Ames test. In the present study, we selected 12 quinoline analogues containing one or two nitrogen atoms in the naphthalene ring and 12 indole analogues containing one to three nitrogen atoms in the indole ring, without any side moiety.

**Results:**

The Ames test was performed with five standard bacterial strains (TA100, TA1535, TA98, TA1537, and WP2*uvrA*) using the pre-incubation method with and without rat liver S9. Five quinoline and two indole analogues were mutagenic. Among the five quinoline analogues, four were mutagenic in the presence of S9 mix with TA100, whereas cinnoline was mutagenic in the absence of S9 mix with TA1537. Among the two indole analogues, indazole was mutagenic in the presence and absence of S9 mix with WP2*uvrA* and 4-azaindole was mutagenic in the absence of S9 mix with TA1537. The mechanisms underlying the induction of mutagenesis appear to differ between quinoline and indole analogues. In addition, we performed in silico analysis of the mutagenicity of all these analogues using DEREK Nexus 6.1.1 (Lhasa Limited) and GT_EXPERT from CASE Ultra 1.8.0.5 (MultiCASE Inc.) as knowledge-based models and GT1_BMUT from CASE Ultra 1.8.0.5 (MultiCASE Inc.) as a statistical-based model. The knowledge-based model showed low sensitivity for both the quinoline and indole analogues (DEREK Nexus and GT_EXPERT: 20% for quinolines and 0% for indoles). Conversely, the statistical model showed high sensitivity (100% for both quinolines and indoles) and low specificity (43% for quinolines and 10% for indoles).

**Conclusion:**

Based on the Ames test results, we proposed structural alerts noting that quinoline analogues were mutagenic when they had nitrogens in any of the positions 2, 5, 7, or 8 in addition to 1, and indole analogues were mutagenic when they had nitrogens at positions 2 or 4 in addition to 1.

## Introduction

The Ames test (bacterial mutagenicity test) is globally used as a gold standard method to detect mutagenicity of chemicals [1–5]. The in silico predictive ability of the Ames test has recently made remarkable progress, reaching a practical level. In fact, in silico evaluation of mutagenicity has become accepted for regulatory applications (*e.g.,* The International Council for Harmonisation of Technical Requirements for Pharmaceuticals for Human Use (ICH)-M7 guideline for pharmaceutical impurities) [6]. However, the predictive ability of some chemical classes is not sufficient owing to a lack of Ames test data [7]. In addition, a lot of the previous data were derived from experiments, where the experimental conditions did not fully meet the recommendation stated in the guidelines, such as the use of five standard bacterial strains and treatment both in the presence and absence of S9 mix. These chemical classes include simple-structured nitrogen atom(s)-containing heterocyclic compounds, which are often used as basic structural moieties in pharmaceuticals.

Some heterocycles are mutagenic in the Ames test and the presence or absence of mutagenicity varies among analogues that differ in the number and position of nitrogen atoms present in the aromatic rings [8]. Quinoline and indole analogues are examples of such chemicals (Table 1). As shown in Table 1, the number of analogues tested is limited and the bacterial strains used in those studies do not completely meet the guidelines for bacterial mutagenicity, which could lead to missed detection of mutagens [7, 9–16]. In addition, the mechanisms underlying heterocycle mutagenesis remain unclear. Therefore, collection of Ames test data for such analogues in the presence and absence of S9 mix with five standard bacterial strains, as recommended in the guidelines, will help to better understand the mutagenic characteristics and contribute to in silico prediction of Ames test results.

**Table 1:** Reported Ames test data

Chemical name		Ames test result	Test strains used	Reference
Quinoline analogues				
Quinoline		Mutagenic (+ S9) in TA100, TA98, WP2*uvrA*	TA100, TA1535, TA98, TA1537, WP2*uvrA* (± S9)	[9]
Isoquinoline		Non-mutagenic	TA100, TA98 (± S9)	[10]
Quinoxaline		Mutagenic (+ S9) in TA98	TA100, TA98, TA102 (± S9)	[7]
		Non-mutagenic	TA100, TA1535, TA98, TA1537, TA1538^*a*^	[7]
Phthalazine		Non-mutagenic	TA100, TA98 (± S9)	[11]
Indole analogues				
Indole		Mutagenic^*b*^	WP2, WP2*uvrA*/pKM101, WP2*uvrA*, TA102 (± S9)	[12]
		Non-mutagenic	TA100 (-S9)	[13]
		Non-mutagenic	TA100, TA98 (-S9)	[14]
Indazole		Non-mutagenic	TA100, TA98 (-S9)	[14]
Benzimidazole		Weakly mutagenic in *HisG46* and TA1530 (-S9)^*c*^	*HisG46*, TA1530, TA1531, TA1532, *HisD3052*, TA1534 (-S9)	[15]
		Non-mutagenic	TA100, TA98 (-S9)	[14]
		Non-mutagenic	TA100, TA1535, TA98, TA1537, TA97 (± S9)	[16]

^a^the presence or absence of S9 mix was not clearly indicated

^b^test strain showed mutagenic was not available

^c^conducted by a spot test

In this study, for investigating the structure-mutagenicity relationships, aiming at improvement of in silico mutagenicity prediction, we performed the Ames test (preincubation method) for 24 simple-structured nitrogen atom(s)-containing heterocyclic compounds (12 quinoline and 12 indole analogues), without any side moiety. Ames test data on the 17 compounds among them have not been reported before. Moreover, the data available even for seven compounds were tested using some of the five standard bacterial strains (only quinoline was tested with all strains). We also conducted in silico analysis of these compounds for Ames mutagenicity, using DEREK Nexus (Lhasa Limited) and CASE Ultra GT_EXPERT (MultiCASE Inc.) as knowledge-based models and CASE Ultra GT1_BMUT (MultiCASE Inc.) as a statistical-based model. Based on the Ames test data obtained, we propose structural alerts for the quinoline and indole analogues, without any side moiety.

## Materials and methods

### Materials

**Fig. 1:**
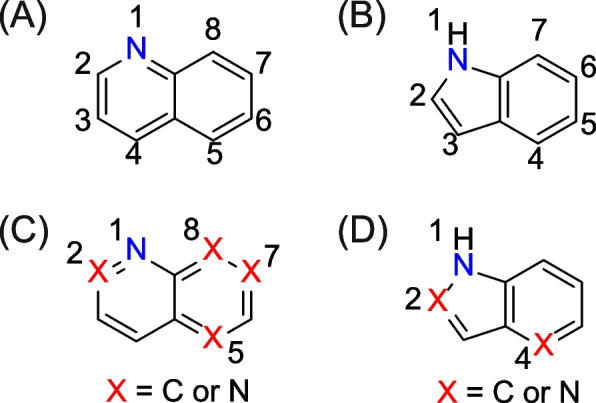
Chemical structures and position number of (**A**) quinoline and (**B**) indole. Suggested structural alerts for (**C**) quinoline and (**D**) indole analogues

Twelve quinoline and twelve indole analogues were tested as test chemicals in this study (Table 2). In the present study, the quinoline analogues contained one or two nitrogen atoms and the indole analogues contained one to three nitrogen atoms in the aromatic ring (Fig. 1A and B). Table 2 lists the chemical name, CAS registry number (CAS No.), source, and purity of test chemicals.

**Table 2:** Chemical name, CAS No., source and purity

Chemical name (synonym)		CAS No	Source	Purity (%)
Quinoline analogues				
Quinoline		91–22-5	Tokyo Chemical Industry	98.9
Isoquinoline		119–65-3	Tokyo Chemical Industry	97.8
Cinnoline		253–66-7	Ambeed	> 95
Quinazoline		253–82-7	Tokyo Chemical Industry	100
Quinoxaline		91–19-0	Tokyo Chemical Industry	99.9
1,5-Naphthyridine		254–79-5	Tokyo Chemical Industry	99.9
1,6-Naphthyridine		253–72-5	Tokyo Chemical Industry	98.7
1,7-Naphthyridine		253–69-0	Princeton BioMolecular Research	> 95
1,8-Naphthyridine		254–60-4	Tokyo Chemical Industry	98.2
Phthalazine		253–52-1	Tokyo Chemical Industry	100
2,6-Naphthyridine		253–50-9	Enamine	100
2,7-Naphthyridine		253–45-2	Enamine	100
Indole analogues				
Indole		120–72-9	Tokyo Chemical Industry	100
Indazole		271–44-3	Tokyo Chemical Industry	100
Benzimidazole		51–17-2	Tokyo Chemical Industry	100
4-Azaindole (1*H*-Pyrrolo[3,2-*b*]pyridine)		272–49-1	Tokyo Chemical Industry	99.9
5-Azaindole (1*H*-Pyrrolo[3,2-*c*]pyridine)		271–34-1	Tokyo Chemical Industry	100
6-Azaindole (1*H*-Pyrrolo[2,3-*c*]pyridine)		271–29-4	Apollo Scientific	99.82
7-Azaindole (1*H*-Pyrrolo[2,3-*b*]pyridine)		271–63-6	Tokyo Chemical Industry	99.8
Imidazo[1,2-*a*]pyridine		274–76-0	Tokyo Chemical Industry	99.4
Pyrazolo[3,4-*b*]pyridine		271–73-8	Tokyo Chemical Industry	100
7-Deazapurine (7*H*-Pyrrolo[2,3-*d*]pyrimidine)		271–70-5	Tokyo Chemical Industry	99.8
5*H*-Pyrrolo[3,2-*d*]pyrimidine		272–50-4	Ambeed	98
Imidazo[1,2-*b*]pyridazine		766–55-2	Tokyo Chemical Industry	99.1

The S9 fraction prepared from the liver of phenobarbital/5,6-benzoflavone-pretreated male Sprague–Dawley rats was purchased from Oriental Yeast (Tokyo, Japan). The S9 mix (0.5 mL) consisted of 0.05 mL of the S9 fraction and 0.45 mL of a cofactor solution (Cofactor-1®; Oriental Yeast Co., Ltd.), and contained 8 mM MgCl_2_, 33 mM KCl, 5 mM glucose-6-phosphate, 4 mM NADPH, 4 mM NADH and 100 mM sodium phosphate (pH 7.4).

### Bacterial tester strains

Four strains of *Salmonella typhimurium,* TA100, TA1535, TA98, and TA1537 and one strain of *Escherichia coli,* WP2*uvrA* were used. These bacterial strains are recommended for use in bacterial mutagenicity testing by the Organisation for Economic Cooperation and Development (OECD) test guideline 471 [4] and other guidelines on genetic toxicology in variety of fields, including ICH S-2 [5].

### Ames test

Ames test was conducted using the preincubation method [17, 18]. Frozen working culture stocks of each strain were inoculated into a conical flask containing nutrient broth medium (2.5% w/v; Oxoid Nutrient Broth No.2, Hampshire, UK) and then cultured with a shaking at 37 °C to obtain bacterial cells in the early stationary phase. The cell density of each culture was confirmed to be > 1 × 10^9^ cells/mL. For the tests carried out in the absence of S9 mix, 0.1 mL of the negative control (vehicle), test chemical solution at various concentrations, or positive control solution was added to a test tube, to which 0.5 mL of 100 mM sodium phosphate buffer (pH 7.4) and 0.1 mL of bacterial culture were added. For the tests carried out in the presence of the S9 mix, the S9 mix was added instead of the phosphate buffer. After mixing, the test tubes were preincubated at 37 °C for 20 min in a shaking water bath. After completion, the treatment mixture was immediately added and mixed with 2 mL of 0.05 mM L-histidine/0.05 mM biotin/0.05 mM L-tryptophan molten top agar and the content was poured onto a plate of minimal-glucose agar medium (Tesmedia®, Oriental Yeast). The plates were incubated at 37 °C for approximately 48 h and the revertant colonies that appeared were counted. The sign of the bacterial background lawn was examined as an indicator of cytotoxicity. In addition, the presence or absence of precipitates from the test chemicals was evaluated.

Multiple tests (dose-finding, main, or confirmatory tests) were conducted. The dose-finding test for almost all the test chemicals was conducted with TA100 in the presence and absence of the S9 mix in a single plate per dose. The main test was conducted in duplicate with the five strains in the presence and absence of the S9 mix. In the confirmatory test, all chemicals were tested in duplicate or triplicate except for eight chemicals that showed clearly negative in the main test. Dimethyl sulfoxide (DMSO) was used as a negative control (vehicle).

Positive controls were included in all of the main and confirmatory tests in order to verify that the test system employed worked well; a positive response was found within the respective historical control value (data is not shown).

Mutagenicity was evaluated according to the so-called “two-fold” rule for TA100, TA98, and WP*2uvrA,* or “three-fold” rule for TA1535 and TA1537 [19]. The test chemical was judged as positive (mutagenic) if the following criteria were satisfied: (1) the maximum number of revertants was two or three-fold or more relative to the negative control, (2) a dose-dependent increase in the number of revertants was observed, and (3) the results were reproducible between each test. Historical negative control counts were also considered during the evaluation.

The maximum mutagenic activity (rev/mg) was used as an indicator of the magnitude of mutagenicity among those calculated according to the following equation from each representative experiment (main test or confirmatory test) for chemicals that were judged positive: Mutagenic activity (rev/mg) = (number of revertant colonies at each dose—number of revertant colonies for the negative control) / dose (mg).

### In silico analyses

All chemicals were analyzed using Derek Nexus (knowledge-based model [ver. 6.1.1; Lhasa Limited, Leeds, UK]), CASE Ultra GT_EXPERT (a knowledge-based model, ver. 1.8.0.1.16392.500), and CASE Ultra GT1_BMUT (statistics-based model, ver. 1.8.0.1.11479.500) [CASE Ultra ver. 1.8.0.5; MultiCASE Inc., OH, USA]. The sensitivity and specificity for predictivity were calculated using the following equations:

$$\mathrm{Sensitivity}\;\left(\%\right)=\left(\mathrm{number}\;\mathrm{of}\;\mathrm{compounds}\;\mathrm{predicted}\;\mathrm{to}\;\mathrm{be}\;\mathrm{positive}\;\mathrm{by}\;in\mathit\;silico\right)/\left(\mathrm{number}\;\mathrm{of}\;\mathrm{Ames}-\mathrm{positive}\;\mathrm{compounds}\right)\times100$$

$$\mathrm{Specificity}\;\left(\%\right)=\left(\mathrm{number}\;\mathrm{of}\;\mathrm{compounds}\;\mathrm{predicted}\;\mathrm{to}\;\mathrm{be}\;\mathrm{negative}\;\mathrm{by}\;in\mathit\;silico\right)/\left(\mathrm{number}\;\mathrm{of}\;\mathrm{Ames}-\mathrm{negative}\;\mathrm{compounds}\right)\times100$$

## Results and discussion

**Fig. 2:**
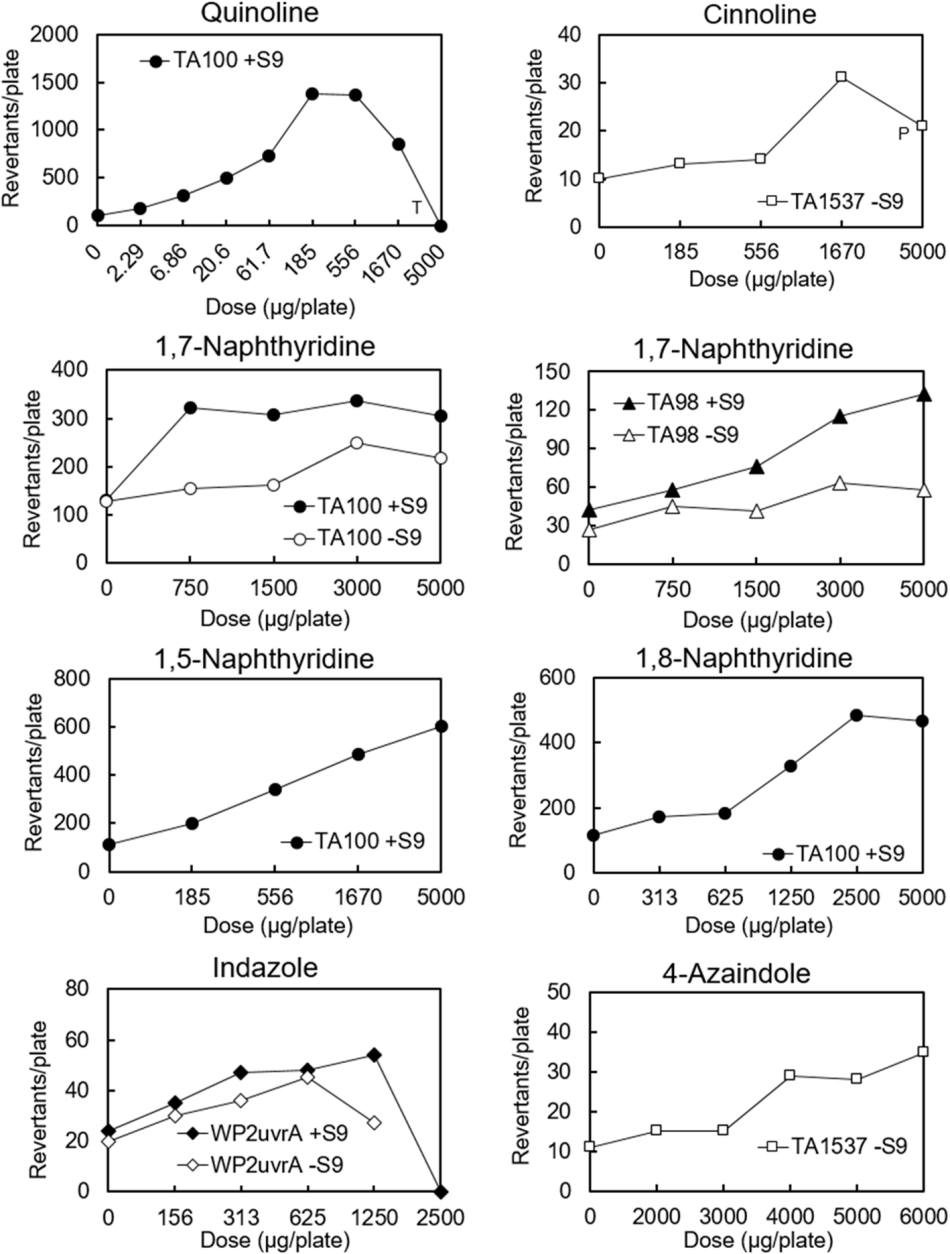
Dose–response curves for the quinoline and indole analogues that were mutagenic. The mean values of revertant colonies/plate in duplicate or triplicate tested in the main or confirmatory test were plotted. The symbols “T” and “P” indicate toxicity (growth inhibition) and precipitation, respectively

Tables 3 and 4, respectively, represent the Ames test results and mutagenic activity of the 12 quinoline (naphthalene substituted with one nitrogen atom at position 1), and 12 indole (indene substituted with one nitrogen atom at position 1) analogues tested with the five standard bacterial tester strains in the presence and absence of the S9 mix. The dose–response curves for each positive compound are shown in Fig. 2.

**Table 3:**
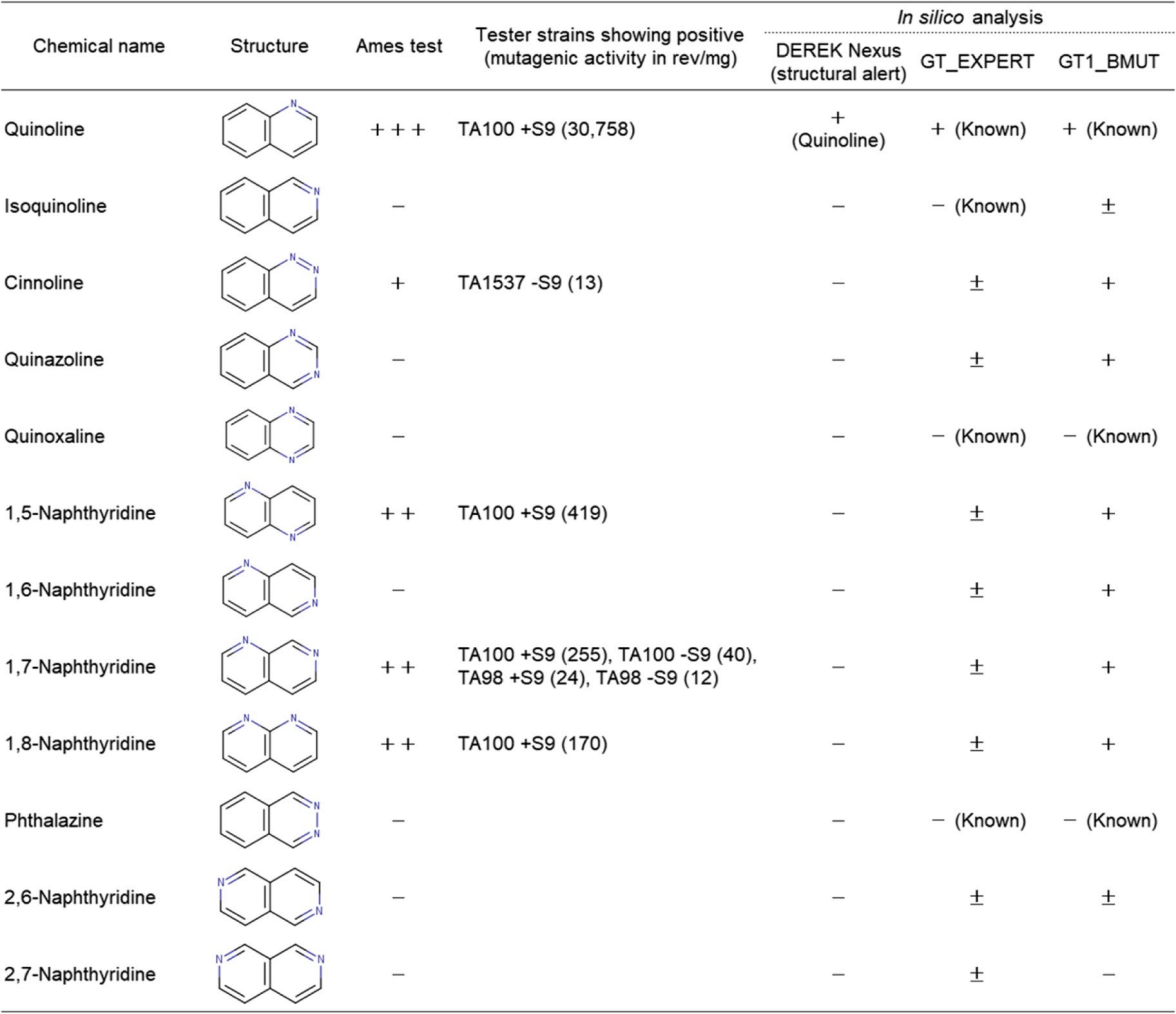
Results of Ames test and in silico analyses of quinoline analogues

+++: "mutagenic (>1000 revertants/mg)" in Ames test

++: "mutagenic (100-1000 rev/mg)" in Ames test

+: "mutagenic (< 100 revertants/mg)" in Ames test, "positive" in CASE Ultra and "probable" in Derek Nexus

±: "inconclusive" in CASE Ultra and "equivocal" in Derek Nexus

-: "non-mutagenic" in Ames test, "negative" in CASE Ultra, "inactive" in Derek Nexus

DEREK version: Derek Nexus: 6.1.1, Nexus: 2.4.0, CASE Ultra Version: 1.8.0.5

**Table 4:**
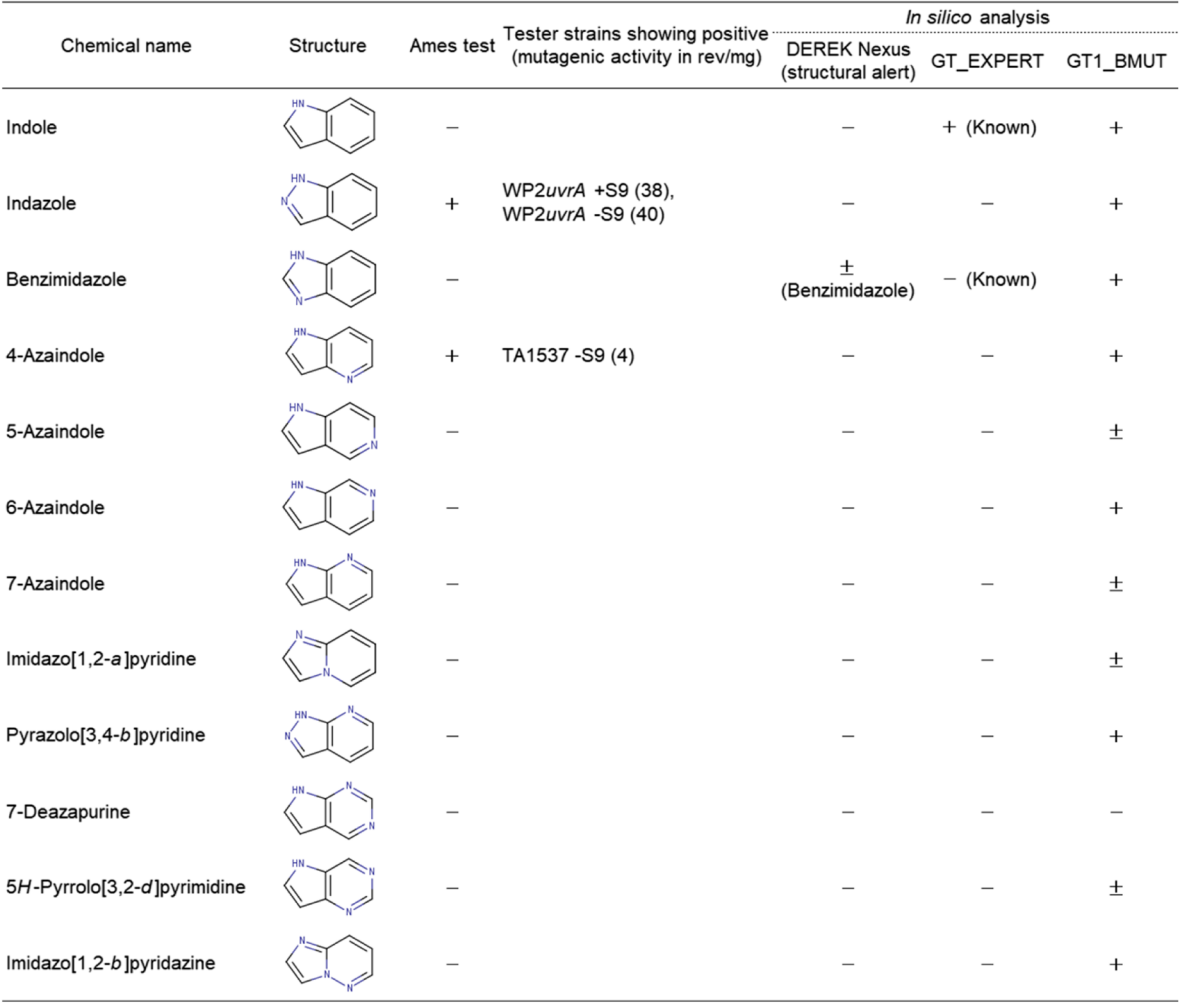
Results of Ames test and in silico analyses of indole analogues

+: "mutagenic (< 100 revertants/mg)" in Ames test, "positive" in CASE Ultra and "probable" in Derek Nexus

±: "inconclusive" in CASE Ultra and "equivocal" in Derek Nexus

-: "non-mutagenic" in Ames test, "positive" in CASE Ultra, "inactive" in Derek Nexus

DEREK version: Derek Nexus: 6.1.1, Nexus: 2.4.0, CASE Ultra Version: 1.8.0.5

Tables 3 and 4, respectively, also represent the results using in silico analysis tools. It is important to note that validation of the in silico tools against a small data set is limited in this study.

### Ames test results of quinoline analogues

Five of the twelve quinoline analogues tested were mutagenic. In the mutagenic quinoline analogues, quinoline, naphthalene substituted one nitrogen atom at position 1, had the highest mutagenic activity [30,758 rev/mg (TA100, + S9)], followed by 1,5-naphthyridine [419 rev/mg (TA100, + S9)], 1,7-naphthyridine [255 rev/mg (TA100, + S9)], 1,8-naphthyridine [170 rev/mg (TA100, + S9)]), and cinnoline [13 rev/mg (TA1537, -S9)].

Quinoline, 1,5-naphthyridine, and 1,8-naphthyridine were only mutagenic in TA100, which primarily detected a base-pair substitution mutation at the G-C base pair. 1,7-Naphthyridine was mutagenic in both TA100 and TA98 that detects frameshift mutations at the G-C base pair. Cinnoline, with nitrogen atoms at positions 1 and 2, was mutagenic in TA1537, that detects frameshift mutations at the G-C base pairs, a different type of frameshift mutation from TA98. Thus, four of the five mutagens were detected by TA100 and two by either TA98 or TA1537, indicating that quinoline analogues form DNA adducts at G-C base pairs, which mainly induce base-pair substitution mutations and some frameshift mutations, respectively.

Quinoline, 1,5-naphthyridine, and 1,8-naphthyridine were mutagenic only in the presence of the S9 mix, whereas 1,7-naphthyridine was mutagenic both in the presence and absence of the S9 mix. Cinnoline was mutagenic only in the absence of the S9 mix. The four compounds that were mutagenic in the presence of the S9 mix had one nitrogen atom in the ring at position 1. The mutagenicity of quinoline is suggested to be attributable to the formation of an enamine epoxide, 2,3-epoxide of 1,4-hydrated quinoline (enamine-epoxide theory) [20]. Therefore, the mutagenicity of these compounds may be initiated by the epoxidation of the drug-metabolizing enzymes present in the S9 mix. The finding that quinazoline and quinoxaline, quinolines substituted one nitrogen atom at position 3 or 4, respectively, were non-mutagenic suggests that nitrogen atoms substituted at the positions inhibit epoxidation for metabolic activation. Isoquinoline, phthalazine, 2,6-naphthyridine, and 2,7-naphthyridie that did not substitute nitrogen atom at position 1 in the naphthalene ring, were non-mutagenic. This observation suggests that the nitrogen atom at position 1 is essential for metabolically activating the mutagenicity of quinoline analogues. 1,6-Naphthyridine was not mutagenic although it contained a nitrogen atom at position 1. The nitrogen atom at position 6 might inhibit metabolic activation. Reasons of mutagenicity of 1,7-naphthyridine and cinnoline in the absence of the S9 mix is unclear. Since these chemicals are chemically stable, we have speculated that their mutagenicity was induced by reactive metabolite(s) produced by the bacterial oxidases present in cells via a different mechanism from the enamine-epoxide theory [20].

### In silico analysis of quinoline analogues

The DEREK Nexus (knowledge-based model) predicted only quinoline to be mutagenic (probable called) and other non-mutagenic (inactive called), as shown in Table 3. The remaining four mutagens were predicted to be non-mutagenic. Therefore, the sensitivity was 20% (1/5 mutagens) and DEREK Nexus was shown to have a low sensitivity for the mutagenicity prediction of quinoline analogues because it had only a quinoline alert for these analogues. Conversely, the specificity was as high as 100% (7/7 non-mutagen).

**Fig. 3:**
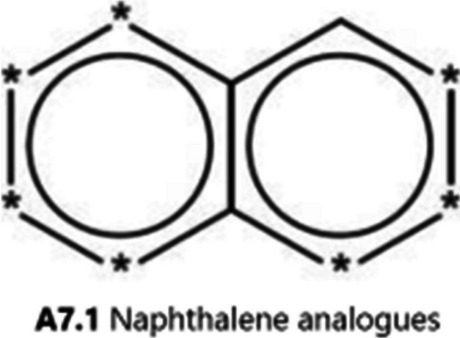
"Naphthalene analogues” structural alert indicated by GT_EXPERT

The sensitivity of another knowledge-based model, GT_EXPERT (CASE Ultra), was 20% (1/5 mutagens), the same as for DEREK Nexus. This model called mutagenic quinoline as known positive and non-mutagenic isoquinoline, quinoxaline, and phthalazine as known negative. Mutagenic cinnoline, 1,5-naphthyridine, 1,7-naphthyridine, and 1,8-naphthyridine were called to be inconclusive, with a probability (59%) of the presence of the “naphthalene analogues” alert (Fig. 3), which was between the cut-off values for positive and negative predictions (40–60%).

GT1_BMUT (CASE Ultra statistical-based model) predicted the five mutagenic analogues (quinoline, cinnoline, 1,5-naphthyridine, 1,7-naphthyridine, and 1,8-naphthyridine) and three non-mutagenic analogues (quinazoline, quinoxaline, and 1,6-naphthyridine) as positive. Thus, the sensitivity was 100% (5/5 mutagens). For the four non-mutagenic quinoline analogues substituted with nitrogen atom not at position 1, isoquinoline and 2,6-naphthyridine were called to be inconclusive, and 2,7-naphthyridine and phthalazine negative. Thus, the specificity was as low as 43% (3/7 non-mutagens), based on the structural alert of quinoline.

### Ames test results of indole analogues

Among the 12 indole analogues, indazole and 4-azaindole were mutagenic, whereas the other 10 were not (Table 4).

Indazole was weakly mutagenic in the presence of S9 mix (38 rev/mg) and in the absence of S9 mix (40 rev/mg) with WP2*uvrA* that detected base-pair substitution mutations at the A-T base pairs. Since indazole showed a similar dose–response curve between the presence and absence of S9 mix, it was considered a direct mutagen that does not require enzymatic activation for mutation induction. 4-Azaindole was also weakly mutagenic in the absence of the S9 mix (4 rev/mg) with TA1537, that detected frameshift mutations in the G-C base pairs. The reason for mutagenicity of indazole and 4-azaomdole in the absence of S9 mix is unclear. These compounds were chemically stable. Therefore, we speculated that their mutagenicity was attributable to reactive metabolite(s) produced by bacterial oxidases.

Indole was not mutagenic. Indazole with a nitrogen atom at position 2 of indole ring, was mutagenic, while pyrazolo[3,4-*b*]pyridine with two nitrogen atoms at positions 2 and 7 of indole ring, was non-mutagenic. 4-Azaindole, indole substituted one nitrogen atom at position 4 was mutagenic, whereas 5*H*-pyrrolo[3,2-*d*]pyrimidine with two nitrogen atoms at positions 4 and 6 of indole ring was non-mutagenic. Therefore, the presence of one nitrogen atom at either position 2 or 4 of the indole ring results in mutagenicity. However, the introduction of neither one nitrogen atom not at position 2 or 4 nor two nitrogen atoms in the indole ring, induces mutagenicity.

### In silico analysis of indole analogues

Both indazole and 4-azaindole were mutagenic. Benzimidazole was not mutagenic in this study; however, it has been reported as weakly-mutagenic (with adjunct strains, *Salmonella typhimurium HisG46* and TA1530 by a spot test) or non-mutagenic [15, 16]. Because of the conflicting data, DEREK Nexus called benzimidazole equivocal. DEREK Nexus predicted the mutagenicity of the indole analogues with no sensitivity (0/2 mutagens), since it had no alert(s) for these analogues, except for the structural alert of benzimidazole. Accordingly, DEREK Nexus predicted nine out of ten non-mutagens (specificity = 90%).

Another knowledge-based model, GT_EXPERT, referred to indole as known positive, benzimidazole as known negative, and ten others as negative. Indazole and 4-azaindole, which were mutagenic in this study, were not predicted to be positive with no sensitivity (0/2 mutagens). Contrarily, indole, which was non-mutagenic in the present study, was called as known positive [12]. GT_EXPERT showed no structural alerts except for exact match for indole analogues and predicted nine out of ten non-mutagens as negative (specificity = 90%).

The GT1-BMUT (statistical model) predicted seven analogues as positive, four analogues as inconclusive, and one analogue as negative, with each indole analogue having slightly different alert(s) providing different predictions of positive or inconclusive with high sensitivity of 100% (2/2 mutagens) and low specificity of 10% (1/10 non-mutagens).

### Proposed structural alerts for quinoline and indole analogues

The goal of our studies is to firstly collect Ames test data for compounds which are often used as basic structural moieties in pharmaceuticals, but do not have enough number of reliable Ames test data, using the five guideline-recommended strains for determining mutagenic characters, and thereby improving the Ames mutagenicity prediction up to a practical level (> 75%).

For the quinoline and indole analogues, both knowledge-based models showed many false negative results (low sensitivity) with the structural alert of quinoline or naphthalene analogues, while the statistical-based model showed many false positive results (low specificity) with the alert fragments derived from the limited number of mutagenic compounds, quinoline and indole.

For improvement of in silico predictive ability, we propose the structural alerts specific for the quinoline and indole analogues, based on the Ames test results, as shown in Fig. 1(C) and (D). Figure 1(C) shows that the structural alert for the quinoline analogues, where nitrogen is substituted at positions 2, 5, 7 or 8, or not (quinoline) in addition to position 1 of the naphthalene ring. Figure 1(D) shows that the structural alert for the indole analogues, where nitrogen is substituted at positions 2 or 4 in addition to position 1 of the indole ring, excluding indole. Both structural alerts provide 100% accuracy in predicting mutagenicity of the quinoline and indole analogues without any side moiety, compared to DEREK Nexus and two prediction models of CASE-Ultra.

## Conclusion

We performed the Ames test for 12 quinoline and 12 indole analogues with five standard bacterial tester strains in the presence and absence of the S9 mix to determine their structure-mutagenicity relationships with the proposed structural alerts for quinoline and indole analogues, without any side moiety. Almost half of the quinoline analogues were mutagenic, requiring metabolic activation by the S9 mix for most of them, whereas only a few indole analogues were mutagenic. In silico analyses of both analogues showed that the statistical model predicted the Ames results with high sensitivity but low specificity as it identified multiple structural alerts for both analogues in the query compounds. However, both knowledge-based models predicted the Ames results of the quinoline and indole analogues with low sensitivity as they had no structural alerts appropriate for these chemical classes. The proposed structural alerts allow us to reliably predict the mutagenicity of quinoline and indole analogues without any side moiety.

In silico Ames prediction has become increasingly practical in recent years. However, some chemical classes with low prediction remain because of the lack of Ames data obtained from experiments that were performed using the standard experimental methods, as exemplified in this study. The collection of Ames data for these chemical classes is important for further improvement of in silico predictions [21].

### Future perspective

In this study, the effect of the substituents on mutagenicity was not examined. When substituent(s) with and/or without structural alert(s) (*e.g.,* nitro group, amino group, or alkyl halide) are attached to the quinoline or indole ring, its mutagenic potential can vary depending on the substituents, including the oxidative metabolites of nitrogen-containing heterocycles, such as aromatic *N*-oxides. Data on the mutagenicity and mechanisms of mutation induction are still limited. Therefore, further studies are required.

## Data Availability

The data generated and analyzed during the current study are available from the corresponding author on reasonable request.
